# L’incidence des infections nosocomiales urinaires et des sites opératoires dans la maternité de l’Hôpital Général de Référence de Katuba à Lubumbashi en République Démocratique du Congo

**DOI:** 10.11604/pamj.2017.28.57.9866

**Published:** 2017-09-21

**Authors:** Hendrick Mbutshu Lukuke, Eric Kasamba, Abdulu Mahuridi, Roger Ngatu Nlandu, Suganuma Narufumi, Abel Ntambue Mukengeshayi, Vicky Malou, Michel Makoutode, Françoise Malonga Kaj

**Affiliations:** 1 Ecole de Santé Publique, Université de Lubumbashi, République Démocratique du Congo; 2 Laboratoires des Cliniques Universitaires de Lubumbashi, République Démocratique du Congo; 3 Medical Sciences Cluster Cooperation Medicine Kochi University, Kochi, Japan; 4 Centre de Formation de Santé Publique, Lomé, République du Togo; 5 Institut Régional de Santé Publique, Ouidah, République du Benin

**Keywords:** Incidence, infection nosocomiale, maternité, Incidence, nosocomial infection, maternity

## Abstract

**Introduction:**

Les patients hospitalisés en Afrique intertropicale sont exposés à un risque des infections nosocomiales. La rareté des données publiées sur le sujet limite l’analyse descriptive de la situation. L’objectif de ce travail était de déterminer l’incidence, les germes en cause et les facteurs de risque des infections nosocomiales urinaires et des sites opératoires à la maternité de l’HGR Katuba de Lubumbashi en République Démocratique du Congo(DRC).

**Méthodes:**

Nous avons réalisé une étude descriptive longitudinale de la période allant du 1^er^ octobre 2014 au 1^er^ Janvier 2015. Notre population d’étude était constituée de 207 femmes ayant séjourné dans cette maternité. La collecte de données était réalisée d’une manière exhaustive.

**Résultats:**

L’incidence de ces infections nosocomiales était de 15,5%. Les parturientes ayant passé plus de trois jours à l'hôpital avaient trois fois plus de risque de développer une infection nosocomiale (p = 0,003) tandis que celles qui ont eu un accouchement avec complication avaient quatre fois plus de risque de contracter une infection nosocomiale (p = 0,000). *Escherichia coli* était l'agent causal le plus isole (38,1%), suivi de *Citrobacter freundii* (23,8%), *Acinobacter baumannii* (18, 2%), Staphylococcus aureus (18,2%), *Enterococcus feacalis* (14,3%) et *Pseudomonas aeruginosa* (9,1%). L'ampicilline était l'antibiotique le plus prescrit auquel tous les microbes isoles ont été résistants.

**Conclusion:**

Il faut améliorer des conditions d’hygiène hospitalière; mais aussi une étude ultérieure pour étudier la ressemblance entre les souches des germes de l’environnement et celles trouvés dans les liquides biologiques.

## Abstract (en)

**Introduction:**

In Intertropical Africa hospitalized patients are exposed to a risk of nosocomial infections. The dearth of published data on this subject limits the descriptive analysis of the situation. This study aimed to determine the incidence, the germs responsible for these infections and the risk factors of nosocomial infections in the Maternity Ward at the General Referral Hospital in Katuba, Lubumbashi, Democratic Republic of the Congo.

**Methods:**

We conducted a descriptive, longitudinal study from 1 October 2014 to 1 January 2015. Our study population consisted of 207 women who had been hospitalized in the Maternity Ward at the General Referral Hospital in Katuba. We carried out a comprehensive data collection.

**Results:**

Nosocomial infection rate accounted for 15.5%. Parturient women who had been hospitalized for more than three days were three times more likely to develop a nosocomial infection (p=0.003), while those who had had a complicated delivery were four times more likely to be at risk of developing nosocomial infection (p = 0.000). Escherichia coli was the most isolated causative agent (38.1%), followed by Citrobacter freundi (23.8%), Acinobacter baumani (.18, 2%), Staphylococcus aureus (18.2%), Enterococcus aureus (14.3%) and Pseudomonas aeroginosa (9.1%). Ampicillin was the most prescribed antibiotic, to which isolated microbes were resistant.

**Conclusion:**

It is necessary to improve hospital hygiene and to conduct further study to examine the similarity between germs strains in the environment and those in biological fluids.

## Introduction

Les hôpitaux sont des milieux qui réunissent de différentes maladies, et constituent un milieu propice pour la propagation de ces infections, lorsque les conditions d’hygiène et environnementales ne sont pas favorables [1]. La promiscuité des malades dans les salles et dans les chambres d’hospitalisation favorise les infections transmissibles. De même, le non-respect des pratiques d’asepsie constitue un risque susceptible d’entrainer l’augmentation de ces infections [1, 2]. Ces infections sont majoritairement causées par des bactéries qui présentent souvent des profils de résistances aux antibiotiques. Cela complique souvent leur prise en charge [3]. Les patients hospitalisés en Afrique intertropicale sont exposés à un risque nosocomial majoré. Les principaux facteurs favorisants sont liés aux conditions d’exercice médical, à l’environnement médical. Dans les pays en développement, la rareté des données publiées sur le sujet limite l’analyse descriptive de la situation [2–4]. Les données sur les infections nosocomiales (IN) sont rares à Lubumbashi alors que ces dernières constituent un problème de santé publique dans le monde [3, 4]. Ce travail effectué à la maternité de l’Hôpital Général de Référence Katuba de Lubumbashi a pour objectifs de déterminer l’incidence, les germes en cause et les facteurs de risque des infections nosocomiales urinaires et des sites opératoires dans cette maternité.

## Méthodes

L’étude était réalisée à la maternité de l’Hôpital Général de Référence de Katuba, il est situé à l’extrême sud-est de la commune urbano-rurale portant le même nom, dans la ville de Lubumbashi en République Démocratique du Congo (RDC).

Nous avons réalisé une étude longitudinale; nous nous sommes intéressés à la proportion des infections nosocomiales chez toute femme qui venait pour un accouchement et qui n’avait pas une infection urinaire à son admission. Le suivi de ces femmes était fait jusqu’ à la sortie de la maternité, tandis que pour les césarisés, c’était jusqu’à 30 jours après la sortie. L’investigation a couvert la période allant du 1^er^ octobre 2014 au 1^er^Janvier 2015 soit une période de trois mois.

La population de notre étude était constituée des femmes ayant séjourné dans cette maternité. Etait incluse dans notre enquête, toute femme qui venait à cette maternité pour raison d’un accouchement et à qui nous avons réalisé la première analyse des urines avant ces 12 heures de séjours d’hospitalisation. Celles qui avaient une infection urinaire à l’admission étaient exclues directement de ce groupe. La collecte de données était réalisée d’une manière exhaustive chez toutes les femmes à l’admission et à la sortie.

Les échantillons des liquides biologiques ont été prélevés et analysés au laboratoire des cliniques universitaires de Lubumbashi (CUL) pour identifier les germes en cause et en suite étudier l’antibiogramme. Nous avions analysés les paramètres sociodémographiques et obstétricaux des parturientes, la survenue d’une infection nosocomiale, les germes en cause de ces infections, la résistance des ces germes aux antibiotiques… Un cas d’infection nosocomiale était défini selon la définition de l’OMS, notamment: « des infections survenant chez un patient au sein de l’hôpital chez qui cette infection n’était ni présente ni en incubation au moment de l’admission pendant la période de notre enquête et faisant partie de notre échantillon». L’infection de site opératoire c’est tout écoulement purulent, abcès ou cellulite extensive sur le site opératoire dans le mois suivant une intervention chirurgicale, tandis que l’infection urinaire c’est l’uroculture positive (une ou deux espèces) avec au moins 105 bactéries/ml, avec ou sans symptômes cliniques. Pour le prélèvement sur la plaie opératoire, nous l’avions réalisé en faisant une rotation de 360° et en couvrant une surface de 1cm^2^

Nous nous sommes servis des écouvillons stériles que nous avions humidifiés dans un liquide stérile isotonique. Ces écouvillons étaient passés sur des zones définies en stries parallèles rapprochées en les faisant tourner légèrement, puis sur les mêmes zones en stries perpendiculaires et à la fin, ils étaient remisent dans ses étuis protecteurs qui portaient toutes les identifications et étaient transmis au laboratoire dans un délai d’un quart d’heure. Les écouvillons étaient ensemencés sur une gélose lactosée au pourpre de bromocresol, milieu Chapman, gélose au sang et gélose chocolat, puis incubés à 37°C durant 24 à 48 heures, l’abondance des colonies était notée et les colonies de bacilles à Gram négatif ont été repiquées sur une galerie d’identification API 20E ou API 20 NE et les colonies Gram Positif ont été identifié selon leur réaction à la catalase, et agglutination sur plaque. Chaque prélèvement a été répété et identifié 3 fois. La détermination de la sensibilité des bactéries aux antibiotiques a été effectuée selon la méthode de *diffusion de Kirby Bauer qui classe les souches en trois catégorie: Sensibles (S)*, Intermédiaire *(I)* et résistantes *(R)* sur base du diamètre critique. Tandis que pour le prélèvement des urines, les parturientes l’ont réalisé eux-mêmes après une petite séance éducation sanitaire. Deux prélèvements ont été réalisés, un à l’entrée et un autre après un séjour hospitalier de plus de 72 heures.

Les données ont été analysées à l’aide du logiciel Epi info version 7 et nous avons fait recours aux statistiques usuelles pour décrire nos échantillons et calculer les mesures de fréquence. L’association entre les facteurs d’expositions potentielles et la survenue des infections nosocomiales étaient recherchée au seuil de signification de 5%. Le respect des principes d’éthique était de rigueur, le protocole de recherche était validé par le comité d’éthique de l’Université de Lubumbashi. Les parturientes qui ont été sélectionnées pour participer à l’enquête ont eu les explications claires pour l’objectif et le déroulement de celle-ci afin d’obtenir leurs consentement éclairé verbal, les informations sur les enquêtés ont été gérées d’une manière discrète en anonymat.

**Figure 1:**
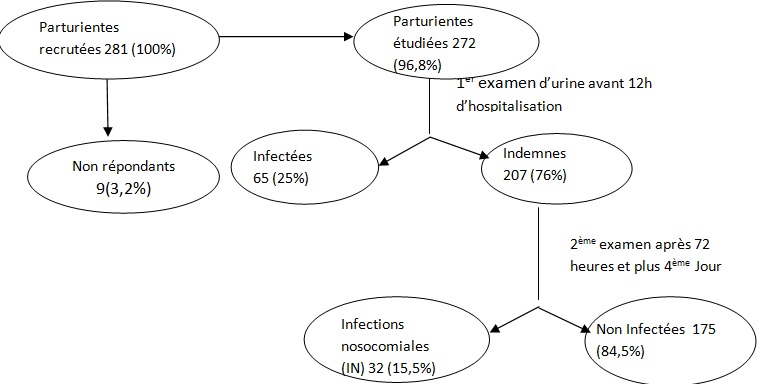
Diagramme du flux d’étude

Au total, nous avons reçu 281 parturientes dont 9 n’ont pas accepté de participer à l’étude en refusant de donner les urines soit à l’entrée ou à la sortie et nous sommes restés avec 272. A la première analyse des urines à l’admission soit avant 12heures de séjours, 65 avaient une infection urinaire et ont été immédiatement exclus de l’étude et nous sommes restés avec 207. A la deuxième analyse des urines au 3jours après hospitalisation et jusqu’ à 30 jours pour les plaies opératoires, 32 soit 15,5% ont développé une infection nosocomiale (Figure 1).

## Résultats

**Données générales:** Les parturientes enquêtées avaient un âge moyen de 27±7,1ans avec un âge minimal de 15ans et maximal de 46ans; 93,7% des accouchés étaient des mariées et 6,3% des célibataires. 84,1% des accouchés avaient un niveau d’étude bas et seulement 15,9% étaient de niveau moyen. La parité moyenne des accouchés était d’environ 3 enfants par femme mais il est noté qu’il y en a aussi celles qui ont eu plus de 7 enfants. L’âge gestationnel moyen à l’accouchement était de 36,6 semaines d’aménorrhée, avec un écart-type de 3,8. La durée moyenne de séjour à la maternité était de 9 jours. Plus de la moitié des accouchés ont eu un accouchement avec complications soit 56,6% dont 47,9% étaient de cas des césariennes; 23,5% d’épisiotomies; 13% des hémorragies; 11,5% d’éclampsie et 4,1% pour les autres dystocies (Tableau 1).

**Tableau 1:** Les paramètres sociodémographiques et obstétriques des parturientes

Age des parturientes, de la grossesse et le mode d’accouchement	Effectif	%
**Tranche d’âge**		
<18	19	10
18-24	67	32
25-31	66	32
32-38	42	20
39 et plus	13	6
**Tranches de la grossesse**		
28-37	107	51,6
38-42	93	44,9
>42	7	3,4
**Mode d’accouchement**		
Accouchement avec complication	123	59,4%
Accouchement sans complication	84	40,6 »%
**Les différentes complications**		
Eclampsie et autres dystocie	19	15,6%
Césarienne	59	47,9%
Episiotomie	29	23,5%
Hémorragie	16	13%

**Figure 2:**
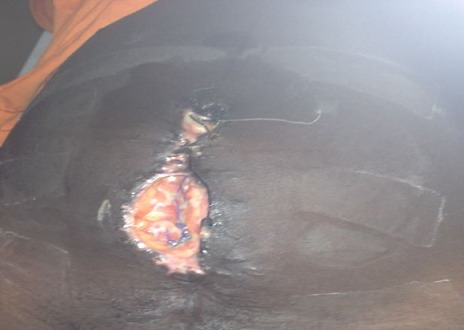
Photo d’un cas d’infection nosocomiale à l’Hôpital général de Référence de Katuba

**L’incidence des infections nosocomiales:** Elle était de 15,5% chez les accouchés avec un intervalle de confiance à 95%. La moitié de ces infections nosocomiales sont les infections urinaires soit (53,1%), 37,5% des infections des sites opératoires et 9,4% avaient une association de ces deux types d’infection. Plus de 3/4 des accouchés ont développé l’infection lors de leur séjour à la maternité (81,3%) (Figure 2).

**Données biologiques:** L’*Escherichia coli* était le plus isolé avec 38,1%, le *Pseudomonas aeruginosa* et *Klebsiella spp*, (23,1%) dans les urines, tandis que dans les liquides des plaies opératoires, c’était toujours *Escherichia coli* (27,3%); *Staphylococcus aureus Citrobacter freundii* et *Acinonobacter baumannii* (18,2%) . *Pseudomonas aeruginosa*, *Enterococcus faecalis* n’avaient que (9,1%) (Tableau 2).

**Tableau 2:** Profil des infections nosocomiales

Les types des infections nosocomiales	Effectif	%
Du site opératoire	8	25,0
Urinaire	21	65,5
Du site opératoire et urinaire	3	9,4
**Moment de la survenue de l’infection**		
En cours d’hospitalisation	23	90 ,6
Après la sortie de l’hôpital	9	9,4
**Les germes en cause des IN isolés dans les urines**		
Enterococcus faecalis	3	14,3
Escherichia coli	8	38,1
Pseudomonas aeruginosa	5	23,1
Klebsiella spp	5	23,1
**Les germes en cause des IN isolés dans liquides des sites opératoires**		
Enterococcus faecalis	1	9,1
Escherichia coli	3	27,3
Acinotobacter baumannii	2	18,2
Pseudomonas aeruginosa	1	9,1
Staphylococcus aureus	2	18,2
Citrobacter freundii	2	18,2

%= Pourcentage

**Profil des antibiotiques:** Il est à noter que 172 accouchés soit 83,1% des accouchés ont été soumises sous antibiotique après l’accouchement. L’ampicilline était le plus prescrit en cas d’accouchement sans complication soit 75,5%. L’association d’ampicilline et gentamycine était couramment prescrit en cas d’accouchement avec complications soit 86,9% des cas (Tableau 3).

**Tableau 3:** Profil d’antibiothérapie chez les parturientes

Prescription d’antibiotiques	Effectif	%
Oui	172	83,1
Non	35	16 ,9
**Antibiotiques prescrits pour les accouchements sans complications**		
Ampicilline	*37*	*75,5*
Amoxicilline et Gentamycine	*3*	*6,1*
Amoxicilline	*9*	*18,4*
**Antibiotiques prescrits pour les accouchements avec complication**		
Ampicilline et Erythromycine	*1*	*0,8%*
Ampicilline et Gentamycine	*107*	*86,9%*
Ampicilline, Gentamycine et Métronidazole	*4*	*3,3%*
Ampicilline, Gentamycine, Métronidazole et Ceftriaxone	*4*	*3,3%*
Ampicilline, Ciprofloxacine, Metronidazole	*4*	*3,3%*
Ceftaxime et Cotrimoxazole	*3*	*2,4%*

%= Pourcentage

**Profil de sensibilité:** Les germes isolés étaient à 100% résistants à l’ampicilline et Amoxicilline; la Gentamycine et le Ciprofloxacine connaissaient aussi quelques résistances de 25% à *Escherichia coli* et à *Klebsiella spp*(Tableau 4).

**Tableau 4:** Réponse des germes isolés dans sites opératoires et les urines à l’antibiogramme

Germes	Gentamicine	Ampicilline & Amoxicilline	Ceftaxime	Ciprofloxacine
*Escherichia coli*	100%S	100%R	100%S	100%I
*Citrobacter freundii*	50%I 50%R	100%R	100%S	50%I 50%R
*Acitobacter baumannii*	100%S	100%R	100%S	100%R
*Staphylococcus aureus*	100%S	100%R	50%S 75%R	100%S
*Enterobacter cloacea*	100%S	100%R	100%S	100%R
*Pseudomonas aeruginosa*	100%I	100%R	100%S	100%I
**Réponse des germes isolés dans urines à l’antibiogramme**				
*Pseudomonas aeruginosa*	100%S	100%R	100%S	60%R et 40%I
*Escherichia coli*	25%R 37,5%I 37,5%S	100%R	100%S	87,5%I et 12 ,5%R
*Klebsiella spp*	40% R et 60%S	100%R	100%S	20%S, 40%I, 40%R
*Enterobacter cloacae*	100%S	100%R	100%S	100%S

R= Résistants, S= Sensibles et I= Intermédiaires, %= Pourcentage

**Facteurs de risque:** Les parturientes qui ont passés plus de trois jours à la l’hôpital avaient trois fois le risque de développer ces infections nosocomiales (p = 0,003); de même celles qui ont eu un accouchement avec complication avaient quatre fois le risque de contracter ces infections nosocomiales (p < 0,000) (Tableau 5).

**Tableau 5:** Analyse des facteurs de risque associés à la survenue des infections nosocomiales

	**Effectifs**	**%**	**RR**	**IC**	**P**
**Incidence des IN**					
Oui	32	15,46%			
Non	175	84,54%			
**Etat Civil**					
Mariée	31	96,88%	0,5	[0,7-3,3]	0,4
Célibataire	1	3,13%			
**Parité**					
Multipare	11	34,38%	1,1	[0,6-2,3]	0,6
Primipare	21	65,63%			
**Niveau d'études**					
Bas	3	9,38%	0,6	[0,2-1,7]	0,2
Moyen	29	90,63%			
**Durée de séjour d’hospitalisation**					
3 Jours					
4 Jours ou plus			3	[1,7-11,4]	0,003
Mode d’accouchement					
Avec complications	29	90,63%	4,4	[1,6-12,1]	0,000
Sans complications	3	9,38%			
Prise d’antibiotiques					
Oui	30	93,75%	2,5	[0,7-10,0]	0,09
Non	2	6,25%			

RR= Risque relatif, IC= Intervalle de Confiance,%= Pourcentage

## Discussion

Cette étude qui avait comme objectifs de déterminer l’incidence, les germes en cause des infections nosocomiales et de déterminer les facteurs de risque des infections nosocomiales urinaires et des sites opératoires dans la maternité de l’hôpital général de référence de Katuba a mis en évidence une incidence de 15,5% dont 65,5% étaient des infections urinaires, 25% des infections postopératoires et 9,4% avaient une association de ces deux types d’infection. L’Escherichia coli était le plus isolé mais également le *Pseudomonas aeruginosa*, le *Klebsiella spp* et d’*Enterococcus faecalis*. Et les facteurs des infections nosocomiales identifiés étaient la durée de séjour et le type d’accouchement.

Pour des raisons financières, nous avons considéré seulement les infections des sites opératoires et les infections urinaires du fait qu’elles sont les plus fréquentes. La durée moyenne de séjour à la maternité était de 9 jours. Plus de la moitié des accouchés ont eu un accouchement compliqué soit 59,4% de cas soit 47,9% étaient des cas de césarienne; 23,5% d’épisiotomie; 13% des hémorragies; 11,5% d’éclampsie.

Le taux des infections trouvé par notre étude était largement supérieur à celui de Samou au Mali 6,7% en 2005 dans le service de chirurgie à l’hôpital du point G [5]; de Coignard B en France 4,3% en 2004 [6]; de Ben en Tunisie 9,03% en 2005 [7]; de Togo au Mali, de 7,4 % en 2009 [8]; de Njimenjen au Gabon, 11% en 2002 [9]; de Hedde-Parison F en France 2,6% [9] et de Njall C 12% en 2013 à Douala au Cameroun [10, 11]. Notons aussi que ces études ont cherché la prévalence et ont inclus tous les types d’infections nosocomiales contrairement à notre étude qui n’a considéré que les infections urinaires et du site opératoire.

Les autres études ont trouvé une prévalence de 7,1% dans les pays industrialisés, et de 10% à 15% dans les pays en voie de développement [2, 12, 13]. Seule l’étude réalisée au service de réanimation polyvalente du CHU HASSAN II, a trouvé une incidence supérieure à celle de notre étude de 38,42%. Ceci se justifie par le nombre de dispositifs invasifs en réanimation [13]. Les autres ont trouvées une faible incidence de 2,9% [14] et (2,6%) [15]. Les infections les plus fréquentes sont les infections urinaires chez les patientes sondées, les infections du site opératoire après une césarienne, les endométrites et les infections de l’épisiotomie [12, 14]; conformément à nos résultats qui approuvent: les infections urinaires (65,5%), de sites opératoires (22%) après césarienne.

Dans notre étude *l’Escherichia coli* était le plus isolé avec 38,1%, le *Pseudomonas aeruginosa* et le *Klebsiella spp*, (23,1%) dans les urines tandis que dans les liquides des plaies opératoires, c’étaient *Escherichia coli*(27,3%); *Staphylococcus aureus Citrobacter freundii* et *Acinonobacter baumannii* (18,2%). *Pseudomonas aeruginosa* et *Enterococcus faecalis* n’avaient que (9,1%). L’étude de Malavaud S et al a trouvé: *Streptococcus pyogenes*, *Escherichia coli, Staphylococcus à coagulase négative, Staphyloccus aureus, Proteus mirabillis, Enterococcus faecalis, Enterobacter aergenes et Klebsiella oxytoca* [12, 14]; ces résultats sont presque similaires aux nôtres.

Dans cet ordre d’idée, une autre étude a trouvé que les germes isolés étaient les bacilles à Gram négatif en tête (86,58%) avec la prédominance du *Pseudomonas aeruginosa* (28,04%). Les Cocci à gram positif représentaient 13,41%, les *Staphylocoques* représentaient 9,7% [3].

Les bactéries liées à l'infection nosocomiales dans l’étude de Njall et alliés étaient en majorité *Echerichia coli, Pseudomonas aeruginosa, Acinetobacter baumannii, Staphylococcus aureus*. Ces résultats sont semblables à ceux trouvés dans notre étude [10]. L’étude menée en Tunisie aux deux centres hospitaliers Habib Bourguiba et Hédi Chaker a trouvé que les infections pulmonaires étaient les plus fréquentes (31,9%), suivies des infections urinaires avec (24,6%), puis les infections du site opératoire avec (11,6%) et les bactériémies-septicémies (10,2%) [14]. Alors dans notre étude, nous n’avons pas pris en comptes les infections pulmonaires.

El Rhazi K. en 2007 au Maroc dans son étude a trouvé que les patients ayant séjourné trois semaines et plus à l’hôpital étaient plus exposés au risque de développer une infection nosocomiale que les patients ayant un séjour de moins de trois semaines [16]. Dans notre étude, les parturientes qui ont passés plus de trois jours à la l’hôpital avaient 3 fois le risque de développer une infection nosocomiale (p = 0,003). Le séjour était un facteur de risque non seulement dans notre étude mais aussi dans celle d’El Rhazi. De même celles qui ont eu un accouchement avec complication avaient 4 fois le risque de contracter une infection nosocomiale (p = 0,005). Le taux de prévalence des infectés était cinq fois plus élevé chez les patients opérés que chez les patients non opérés: 11% versus 2, 2% (p = 0,005) a déclaré El Rhazi K [16].

Par rapport à l’antibiothérapie, 83,1% des accouchés ont été soumises sous antibiotiques après l’accouchement. L’ampicilline était le plus prescrit en cas d’accouchement eutocique soit 75,5%. L’association d’ampicilline et gentamycine était couramment prescrit en cas d’accouchement dystocique soit 86,9% des cas. Tandis que, Fki et collaborateurs ont dit qu’une antibiothérapie a été prescrite chez 60% des patients, l’amoxicilline était l’antibiotique le plus prescrit en première intention (28,3%). Le recours à une monothérapie a été noté dans 65% des cas. La résistance aux antibiotiques a été notée dans 21,6% de l’ensemble des prescriptions [17], contrairement à notre étude qui a trouvé une forte résistance voir de 100% à certains antibiotiques, notamment l’amoxicilline et l’ampicilline. Dans l’étude d’Ayoub, les taux de résistance à la ceftazidime, imipénème, ciprofloxacine et amikacine ont été respectivement 34%, 37,1%, 27,1% et 29,6% [18].

Parlant du profil de sensibilité-résistance des germes les plus isolés aux antibiotiques prescrits aux accouchées, nous avons observé que *l’Escherichia coli* avait une résistance de 100% à l’Ampicilline et à l’Amoxicilline, 25% à la Gentamycine, 12,5% à la Ciprofloxacine, et une sensibilité de 100% à la Ceftaxime, 68,7% à la gentamycine; le *Pseudomonas aeruginosa* avait une résistance de 100% à l’Ampicilline, 60% à la Ciprofloxacine et une sensibilité de 100% à la Ceftaxime, 50% à la gentamycine; le *Staphylocoque aureus* avait une résistance de 100% à l’ampicilline, 50% à la Ceftaxime et une sensibilité de 100% à la Gentamycine et à la Ciprofloxacine, 50% à la Ceftaxime et le *Klebsiella spp* avait une résistance de 100% à l’ampicilline et l’Ampicilline, 40% à la Gentamycine, 20% à la Ciprofloxacine et une sensibilité de 100% à la cefatxime, 60% à la Gentamycine et 40% à la Ciprofloxacine.

Nos résultats rejoignent presque ceux de Mchich au Maroc qui a trouvé que le *Pseudomonas aeruginosa* avait une résistance de 88,8% à la Cefotaxime, 84,2% à la Gentamycine et 43,7% à la Ciprofloxacine; l’*Escherichia coli* 100% résistant à l’association de l’Amoxicilline-acide Clavulanique; le *Klebsiella spp* avait une résistance de 45,4% à la Gentamycine et 58,5% à l’association de l’Amoxicilline-acide Clavulanique, 23% à la Ciprofloxacine et le Staphylocoque aureus était à 62,5% à la Gentamycine, 58,5% à l’association de l’Amoxicilline-acide Clavulanique et 23% à la Ciprofloxacine [19]. L’incidence des infections nosocomiales était très élevée dans notre étude, mais quant aux caractéristiques des germes en cause, ils sont presque identiques pour l’ensemble des études de l’Afrique.

## Conclusion

L’incidence des infections nosocomiales urinaires et des sites opératoires est élevée à l’Hôpital Général de Référence Katuba de Lubumbashi. Elle est de 15,5% dont 3/4 sont urinaires et 1/4 des plaies opératoire. Nous avons isolé *Escherichia coli, le Pseudomonas aeruginosa, le Citrobacter freundii, l’Enterococcus faecalis, l’Acinonobacter baumannii, et Staphylococcus aureus*. L’ampicilline était l’antibiotique le plus couramment prescrit et dont tous les germes avaient déjà développé une résistance en elle. Le long séjour, le mode dystocique étaient les facteurs de risque de ces infections nosocomiales. Ainsi une implication totale de toute la communauté hospitalière et alliés dans la sensibilisation sur les mesures d’hygiène hospitalière pour prévenir ce fléau.

### Etat des connaissances actuelles sur le sujet

- La rareté des recherches sur ce thème des infections nosocomiales dans les pays africains en général et dans notre milieu en particulier;
- Les infections nosocomiales sont majoritairement causées par des bactéries qui présentent souvent des profils de résistances aux antibiotiques;
- L’environnement hospitalier conditions, d’exercice médical et les facteurs individuels sont à l’origine de la survenue de ces infections.

### Contribution de notre étude à la connaissance

- La détermination de l’incidence, les germes en cause et les facteurs de risque des infections nosocomiales à la maternité de l’hôpital général de référence Katuba;
- La connaissance de la résistance des germes aux antibiotiques couramment utilisés dans nos hôpitaux;
- Identification des antibiotiques sensibles aux germes multirésistants pour l’amélioration de la prise en charge de ces infections.

## Conflits d’intérêts

Les auteurs ne déclarent aucun conflit d’intérêts.

## Remerciements

Nos remerciements s’adressent aux autorités de l’Hôpital général de Référence de Katuba pour leur soutien et collaboration à la réalisation de ce travail.

## References

[1] Mbutshu HL, Ntambwe AM, Ngatu RN, Suganuma N, Wembonyama SO, Kabyla I (2013). Gestion des déchets d’activités de soins et entretien des locaux à l’hôpital de référence Jason Sendwe de Lubumbashi, République Démocratique du Congo. Hygiènes..

[2] Simon F, Kraemer P, De Pina JJ, Demortiere E, Rapp C (2007). Le risque nosocomial en Afrique intertropicale - Partie 2: les infections des patients. Med Trop..

[3] Monnet T Les infections nosocomiales: l’importance d’un suivi l'epid'emiologique et de l’identification rapide des bactéries en cause: exemple de quelques techniques de diagnostic permettant cette identification précoce. Thèse..

[4] Gilles B (1998). Infections nosocomiales et environnement hospitalier. Paris : Médecine-sciences Flammarion. ISBN.

[5] Samou F (2005). Les infections nosocomiales dans le service de chirurgie « B » de l’hôpital du point G. Thèse de doctorat Université du Mali..

[6] Coignard B, Lepape A, Talon D, Salomon V, Béatrice T (2006). Guide méthodologique d’aide au signalement du critère 2 Version 8bis..

[7] Ben JN, Bouziri A, Kchaou W, Hamdi A, Mnif K, Belhadj S (2006). Épidémiologie des infections bactériennes nosocomiales dans une unité de réanimation néonatale et pédiatrique. Tnisie Médicale..

[8] Togo A, Coulibaly Y, Keita M, Traore A, Kante L, Diakité I (2009). Infections nosocomiales en chirurgie pédiatrique au Mali. Journal de Pédiatrie et de Puériculture..

[9] Hedde-Parison A, Minchella A, Bastide S, Cornille A, Fatton B, Tayrac R (2013). Infections du site opératoire en chirurgie du prolapsus par voie vaginale. Progrès en Urologie..

[10] Njall C, Adiogo D, Bita A, Ateba N, Sume G, Kollo B (2013). Écologie bactérienne de l'infection nosocomiale au service de réanimation de l'hôpital Laquintinie de Douala, Cameroun. Pan Afr Med J..

[11] Rebaudet S, De Pina JJ, Rapp C, Kraemer P, Savini H (2007). Le risque nosocomial en Afrique intertropicale - Partie 4: prévention. Med Trop..

[12] Makoutodé M (2005). La prévention des infections nosocomiales au CNHU – HKM quels défis? La démarche qualité à l’hôpital..

[13] Qassimi L Epidémiologie des infections nosocomiales en milieu de réanimation à propos de 147 cas. Thèse de doctorat de l’université Sidi Mohammed Ben Abdellah n°40/ 2010..

[14] Malavaud S, Bou-Segonds E, Berrebi A (2003). Les infections nosocomiales chez la mère et l’enfant: à propos d’une enquête d’incidence portant sur 804 accouchements. J Gynecol Obstet Biol Reprod..

[15] Institut d'Hygiène et d'Epidémiologie de Bruxelles (NSIH) Résultats nationaux, Rapport annuel octobre 2000-Juin 2001. Bruxelles 2001..

[16] El Rhazi K, Elfakir S, Berraho M, Tachfouti N, Serhier Z (2007). Prévalence et facteurs de risque des infections nosocomiales au CHU Hassan II de Fès (Maroc). La Revue de Santé de la Méditerranée orientale..

[17] Fki H, Yaïch S, Jdidi J, Karray A, Kassis M, Damak J (2008). Epidémiologie des infections nosocomiales dans les hôpitaux universitaires de sfax: résultats de la première enquête nationale de prévalence de l’infection nosocomiale. Tun Infectiol..

[18] Ayoub Z, Lamia K, Jalel B, Amen AM, Abdelraouef G (2012). Profil épidémiologique et résistance aux antibiotiques des Souches de Pseudomonas aeruginosa isolées au centre de Traumatologie et Grands Brûlés en Tunisie durant trois ans. Tunisie Médicale..

[19] Mchich Anas Les infections nosocomiales à propos de 55 collige au Maroc. Thèse de doctorat, université Cheikh Antadiop de Dakar, 2002.

